# Fertility Issues of Breast Cancer Survivors

**DOI:** 10.6004/jadpro.2012.3.5.2

**Published:** 2012-09-01

**Authors:** Hollis McClellan Misiewicz

**Affiliations:** From Mercy Medical Center, Baltimore, Maryland

## Abstract

In the United States, more women are diagnosed with breast cancer than any other type of cancer. More than 11,000 of these women will be younger than 40, and many of these women will want to have children in the future. A significant number of these young breast cancer patients will require treatment that can cause ovarian failure or premature menopause. Several options do exist for fertility preservation, both standard and investigational. Embryo cryopreservation is the most established intervention. Investigational interventions include oocyte cryopreservation, ovarian tissue transplantation, ovarian suppression with a gonadotropin-releasing hormone agonist, and harvesting of immature follicles with in vitro maturation and cryopreservation. Although pregnancy during cancer treatment is not recommended, pregnancies occurring after completion of therapy have not been linked to increased cancer recurrence. Young women diagnosed with breast cancer need evidence-based information presented in a timely manner in order to make decisions regarding fertility preservation prior to the initiation of treatment. The oncology advanced practitioner must be knowledgeable about fertility preservation options available to these women as well as comfortable with ethical and financial concerns that can arise. The informed practitioner can effectively counsel patients and refer to fertility specialists when appropriate.

In the United States, more women are diagnosed with breast cancer than any other type of cancer. A woman in this country today has a 12% chance of developing breast cancer in her lifetime (National Cancer Institute, 2012). In 2011, more than 11,000 women under the age of 40 were diagnosed with breast cancer. Although incidence rates of breast cancer in premenopausal women have remained stable since 1985, the mortality rate has steadily declined (ACS, 2011). Earlier detection of breast cancer and new modalities for treatment are cited as reasons for this trend. Adjuvant therapy has improved survival, but treatment can lead to amenorrhea, early menopause, and loss of fertility (Gorman, Usita, Madlensky, & Pierce, 2011). Since more women are delaying childbearing until their 30s or later, a greater number of breast cancer survivors are faced with reproductive concerns prior to completing their family (Camp-Sorrell, 2009; Sonmezer & Oktay, 2006).

Infertility secondary to cancer treatment is associated with grief that is accentuated by a loss of choice when patients are not informed of fertility preservation options (Lee et al., 2011). Research has shown that this distress is still significant 10 years after diagnosis (Canada & Schover, 2012). The focus of oncology care has always been treatment of disease. As the number of premenopausal women who are diagnosed with breast cancer grows, health-care practitioners must also be prepared to address concerns related to fertility and premature menopause with appropriate counseling about the options that exist for fertility preservation.

## Effects of Treatment on Fertility

At birth, a female has a fixed number of primordial follicles in the ovaries. At puberty, she has approximately 300,000 oocytes, of which 300 to 500 will mature and be released during the reproductive years. Follicle-stimulating hormone (FSH) and luteinizing hormone (LH) produced in the anterior pituitary gland stimulate proliferation of granulosa cells and trigger ovulation (Camp-Sorrell, 2009). The eggs that remain dormant are susceptible to cell damage from chemotherapeutic agents (Fleischer, Vollenhoven, & Weston, 2011). Important factors in determining whether a chemotherapeutic regimen will result in infertility are the age of the patient, the type of chemotherapy, and the cumulative dose of chemotherapy received (Fleischer et al., 2011; Hulvat & Jeruss, 2009; Sonmezer & Oktay, 2006).

Chemotherapy is thought to cause follicular destruction through apoptosis, the programmed death of a cell stimulated by damage to that cell (Sonmezer & Oktay, 2006). As combinations of chemotherapeutic agents to treat breast cancer continue to evolve, the cumulative effects on fertility remain unclear. For every month of chemotherapy that a woman receives, she could potentially lose 1.5 years of fertility (Schover, 2008). Alkylating agents, a class of chemotherapy drugs often used to treat breast cancer, are potent gonadotoxic agents and can damage primordial follicles at rest as well as during any phase of the cell cycle (Fleischer et al., 2011; Sonmezer & Oktay, 2006). This destruction of follicular reserve results in premature menopause and infertility (Camp-Sorrell, 2009). Estimates of ovarian failure after chemotherapy range from approximately 10% to 81% (Kasum, 2006; Knobf, 2006).

Age is a strong predictive factor of ovarian failure following chemotherapy. Follicles must be present in the ovaries in order for a woman to be fertile. When the number of follicles is reduced to zero, menopause occurs. Younger women have a larger reserve of ovarian follicles and can tolerate more chemotherapy without any short-term effect on fertility (Chasle & How, 2003). A younger woman is more likely to have return of menses; however, this does not necessarily indicate that fertility has been preserved (Sonmezer & Oktay, 2006). Even if ovarian function remains intact, the number of follicles will be diminished, resulting in an early menopause and a narrowed window of opportunity for pregnancy to occur (Fleischer et al., 2011; Knobf, 2006). An older woman with fewer ovarian follicles will experience menopause during chemotherapy (Sonmezer & Oktay, 2006).

Guidelines recommend that premenopausal women with hormone receptor–positive breast cancer receive 5 years of treatment with the selective estrogen receptor modulator tamoxifen, which has been linked to a 31% decrease in annual breast cancer deaths (Hickey, Peate, Saunders, & Friedlander, 2009). Although tamoxifen does not cause ovarian follicular destruction, it has been associated with fetal abnormalities in animal models, so pregnancy is discouraged during this time (Hulvat & Jeruss, 2009). For women whose ovarian reserve has been compromised by previous chemotherapy, this 5-year delay could result in ovarian failure prior to the end of treatment (Sonmezer & Oktay, 2006).

## Options for Fertility Preservation

Current options available to women with breast cancer to protect their fertility after chemotherapy range from well-established standard techniques to investigational interventions (Sonmezer & Oktay, 2006). In vitro fertilization (IVF) with embryo cryopreservation is currently the most effective fertility-assisted intervention for women diagnosed with breast cancer. In order to consider embryo cryopreservation, the breast cancer patient must have a partner or be willing to use donor sperm (Lee et al., 2006). The most useful standard for determining the success of IVF is the cumulative delivery rate per stimulation cycle. Delivery rates of 50% to 60% have been achieved for IVF with cryopreserved embryos (Borini, Cattoli, Bulletti, & Coticchio, 2008).

Oocytes must be obtained before the start of chemotherapy in order to avoid fertilizing a damaged egg. Normally, the preparation for egg harvest begins on day 2 after the start of menses. In cases where it is important to initiate cancer treatment as soon as possible, downregulation of the pituitary with a gonadotropin-releasing hormone analog (GnRH analog) such as buserelin1 or goserelin (Zoladex) can be initiated to minimize delays. This will prevent the premature release of oocytes without having to wait for menstruation to start. The ovarian follicles are then stimulated with gonadotropin injections, either human-derived or synthetic FSH or LH, to promote growth. When the ovarian follicles have grown sufficiently, human chorionic gonadotropin is administered to mature the oocytes and trigger ovulation. At 35 to 36 hours after the administration of hCG, oocytes are collected prior to their release from the ovary (Lee, Jee, Suh, Kim, & Moon, 2012). Once the oocytes are matured, they are retrieved by aspiration with a needle placed in the ovary. This is accomplished vaginally, with the use of ultrasound guidance, while the woman is under sedation. Cancer treatment may be started within 48 hours of egg retrieval.

Standard IVF protocols result in the stimulated follicles producing estradiol at levels 10 times that of normal. Elevated estradiol levels are contraindicated for breast cancer patients, as estradiol has the potential to stimulate the growth of breast cancer cells (Hulvat & Jeruss, 2009). Protocols combining gonadotropins and letrozole (Femara), an aromatase inhibitor, for ovarian stimulation have been shown to prevent increases in estradiol levels while resulting in a yield of oocytes similar to that of standard IVF protocols. The use of letrozole and standard fertility medications for ovarian stimulation is suitable for breast cancer patients (Azim, Costantini-Ferrando, & Oktay, 2008; Hulvat & Jeruss, 2009; Rodriguez-Wallenberg, & Oktay, 2010; Sonmezer & Oktay, 2006). Initial research shows no increase in breast cancer recurrence when this protocol is used for embryo cryopreservation for women with breast cancer (Azim, Costantini-Ferrando, & Oktay, 2008). Madrigrano et al. (2007) found that the time from initial fertility consultation to the initiation of chemotherapy after retrieval of oocytes was an average of 46.8 days. This emphasizes the importance of discussing fertility options in a timely manner to avoid delays in treatment (Hulvat & Jeruss, 2009).

For women who do not have a partner or are unwilling to use donor sperm, oocyte cryopreservation is an alternative option for preserving fertility following chemotherapy. Ovarian stimulation is achieved in the same manner as with embryo cryopreservation, so a timely discussion prior to the initiation of treatment is crucial. Recently, freezing techniques have improved, increasing the survival rate of thawed oocytes; however, the success rate with this method is lower than with embryo cryopreservation (Dunn & Fox, 2009; Hickey et al., 2009; Hulvat & Jeruss, 2009; Mertes & Pennings, 2011). Birth rates per thawed oocyte range from 1.6% to 6% (Hulvat & Jeruss, 2009; Lee et al., 2006). To date, over 900 births have been reported using oocyte cryopreservation followed by IVF (Noyes, Porcu, & Borini, 2009).

One investigational option for fertility preservation is the use of a GnRH agonist to arrest follicular release during chemotherapy. GnRH agonists are thought to decrease the destruction of ovarian follicles by chemotherapy, yet the biologic explanation for this phenomenon is unknown (Badawy, Elnashar, El-Ashry, & Shahat, 2009; Gerber et al., 2011). Current data are inconsistent in supporting the effectiveness of the use of GnRH agonists to protect ovarian function (Badawy et al., 2009; Del Mastro et al., 2011; Gerber et al., 2011; Hartmann, Reimer, & Gerber, 2011; Munster et al., 2012). Research by Recchia et al. (2006) demonstrated improved clinical outcomes with increased recurrence-free and overall survival in women who used a GnRH agonist for fertility preservation. Although it appears that the return of menstrual function may be greater in women receiving a GnRH agonist during chemotherapy, the effect on fertility is less clear, with few reported successful pregnancies (Sonmezer & Oktay, 2006). Spontaneous abortions and elected termination of pregnancies due to Down syndrome have been reported in women who became pregnant after use of a GnRH agonist to preserve fertility following chemotherapy (Oktay & Sonmezer, 2008). Some concern exists that a GnRH agonist might interfere with the efficacy of chemotherapy, particularly with hormone receptor–positive tumors (Hartmann et al., 2011; Hulvat & Jeruss, 2009). The American Society of Clinical Oncology (ASCO) recommends the use of GnRH agonists only in approved clinical trials until more evidence of safety and effectiveness is established (Hulvat & Jeruss, 2009; Lee et al., 2006).

Ovarian cryopreservation and transplantation is another investigational option for the preservation of fertility following cancer chemotherapy. This method does not involve ovarian stimulation so the patient avoids the high levels of estradiol that are observed with embryo and oocyte cryopreservation. Treatment is also less likely to be delayed. For this method of fertility preservation, an ovary is surgically retrieved from the patient prior to the start of chemotherapy. This ovarian cortex is then cut into pieces and frozen. Following completion of cancer treatment, at a time when pregnancy is desired, the frozen tissue can be thawed and then auto-transplanted.

Transplantation can be either orthotopic, within the pelvic cavity, or heterotopic, grafted subcutaneously to the forearm or suprapubic region. The orthotopic technique allows for natural conception but does require the use of general anesthesia during transplantation. Heterotopic transplantation allows for easy monitoring of follicle development for later use in in vitro fertilization, does not require abdominal surgery, and can be performed under local anesthesia (Sonmezer & Oktay, 2006; Zakak, 2009). To date, 13 live births have been achieved using ovarian cryopreservation and transplantation, all with the orthotopic technique (Donnez et al., 2011).

A major concern of ovarian cryopreservation and transplantation for fertility preservation is the possibility of reseeding cancer cells that may exist within the ovaries (Hickey et al., 2009; Hulvat & Jeruss, 2009; Sonmezer & Oktay, 2006). A breast cancer patient who carries the *BRCA1* or *BRCA2* gene mutation has an increased risk of developing ovarian cancer. Prior to initiation of ovarian cryopreservation and transplantation, it is important to ascertain the *BRCA* status of the patient. This procedure should only take place in centers that have the capacity to screen the ovarian tissue for cancer cells (Hulvat & Jeruss, 2009). An alternate strategy for fertility preservation that avoids the risks of autotransplantation is the retrieval of immature oocytes from the ovarian tissue followed by in vitro maturation and freezing (Ata, Chian, & Tan, 2010). Ovarian tissue cryopreservation and transplantation is experimental and should only be offered with full disclosure of the risks in a setting with institutional review board oversight (Zakak, 2009).

Yet another option for fertility preservation is the retrieval of immature oocytes from ovaries without the use of hormonal stimulation followed by in vitro maturation (IVM) and cryopreservation of the mature oocytes. Since ovarian stimulation is not required, high estradiol levels are avoided. The eggs can be retrieved at any stage of the menstrual cycle, and the mean number of days from initial consultation to retrieval of the oocytes is only nine days. This option is not widely used but has resulted in live births in women without cancer. Very few breast cancer survivors have attempted fertility through IVM and, to date, there have been no live births in this population. Although it is still experimental, the retrieval of immature oocytes with in vitro maturation and vitrification presents an alternate strategy to offer breast cancer patients desiring fertility preservation (Huang et al., 2010). The options for fertility preservation for young breast cancer patients are highlighted in Table 1.

**Table 1:**
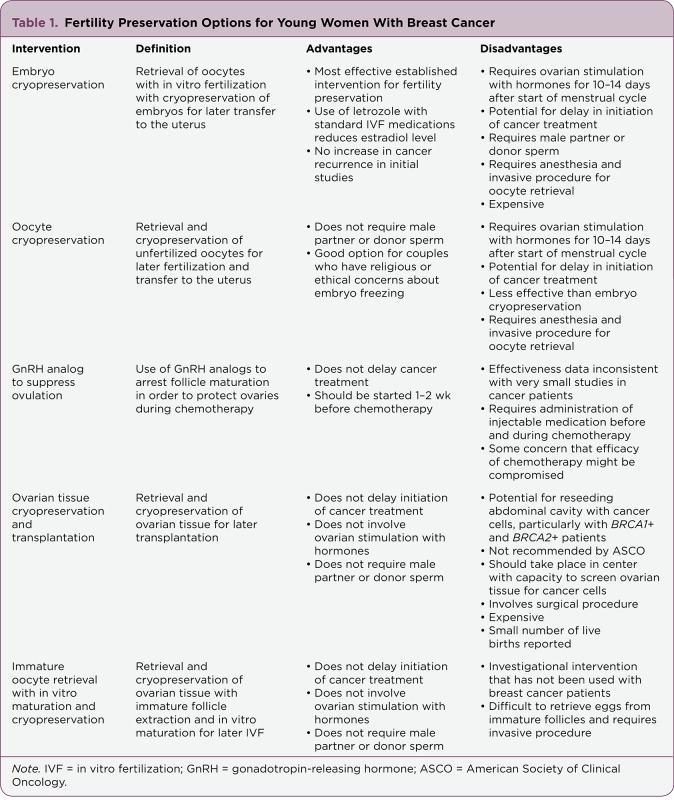
Table 1.Fertility Preservation Options for Young Women With Breast Cancer

## Pregnancy and Breast Cancer Treatment

Although it is possible for women to become pregnant while on chemotherapy, they should be cautioned to avoid doing so. Women might assume that since there is a risk for infertility secondary to cancer treatment, they cannot get pregnant while they are receiving therapy. These patients need to be counseled to use barrier contraceptives during treatment because pregnancy is possible and there is risk of toxicity to oocytes from chemotherapy. Tamoxifen is also linked to teratogenic effects, so pregnancy should be avoided during this therapy as well (Camp-Sorrell, 2009). The optimal time for pregnancy following breast cancer treatment is unknown, as the risk of relapse is related to many factors such as stage of disease, hormone receptor status, and lymph node involvement. Following chemotherapy for breast cancer, it is commonly recommended that women wait 2 years before becoming pregnant, as most cancer recurrences will occur during this time. No benefit has been shown in delaying childbearing longer than this provided the patient has completed adjuvant therapy (Hickey et al., 2009).

The risk of cancer recurrence or death for women with breast cancer who had children following chemotherapy was found to be no greater than the risk for women who did not have a subsequent pregnancy (Kranick et al., 2010). Survival rates in women who become pregnant after cancer treatment are sometimes improved over those seen in women who do not become pregnant (Kasum, 2006). This could reflect a self-selected group of women who may be healthier than women with a higher risk of cancer recurrence who may have chosen to avoid pregnancy (Rodriguez-Wallberg & Oktay, 2010). Breast cancer survivors must be made aware of the fact that the risk for recurrence does exist and could interfere with their ability to care for their children in the future (Hickey et al., 2009; Zakak, 2009).

## Legal and Ethical Issues

Ethical issues can arise when considering the preservation of the fertility of a woman who has been diagnosed with cancer. It is possible that the patient who is cured or in remission following treatment will have a recurrence and die prematurely. The child born after the cancer diagnosis could be left without a mother (Zakak, 2009). While some health-care professionals argue that it might be unethical to assist reproduction in circumstances where a parent might have a short life or be unable to provide care for a child, ethical analysis does not find this stance persuasive. It is perceived that (1) the risk of cancer recurrence varies greatly among patients, (2) a child will have a meaningful life even if he or she experiences the loss of a parent, and (3) although there is a substantial impact on a child from the loss of a parent, children suffer other stresses in life related to economic, social, and physical conditions present in their lives (Ethics Committee of the American Society for Reproductive Medicine [ASRM], 2005).

Another issue involves the disposition of stored tissue or embryos in the event of the patient’s death. At times, some terminally ill patients choose posthumous parenting so that they can leave a legacy for their family (Quinn et al., 2009a; Quinn et al., 2010). The reproductive endocrinologist treating the patient should address this issue prior to the time of tissue storage. The ASRM recommends that a written document be drawn up that specifically states whether the tissue should be discarded upon death of the patient or whether a designee can use the tissue at his or her their discretion. This document would also address issues of ownership of embryos if the oocyte and sperm donors divorced or separated. If this is done properly, legal involvement can be avoided if any of these circumstances occur. Although these issues may be ethically difficult for some health-care professionals to address, the Ethics Committee of the ASRM (2005) recommends assistance for all cancer patients who desire fertility preservation.

## Financial Barriers

Fertility treatments are generally very expensive and frequently not covered by insurance. This can be a significant barrier for patients with breast cancer who would like to proceed with fertility preservation interventions. In vitro fertilization can cost from $6,000 to $12,000 for a single cycle. Medications to stimulate ovulation and storage of frozen embryos or oocytes are additional expenses (Quinn, Vadaparampil, Bell-Ellison, Gwede, & Albrecht, 2008). Fifteen states currently have laws requiring insurance to pay for fertility treatments, although the extent of coverage is variable (Fertility LifeLines, 2012). Cancer patients are often excluded from this insurance coverage, as they are not technically "infertile" at the time they are seeking treatment (Shah, Goldman, & Fisseha, 2011).

Fertile Hope is a national LIVE**STRONG**  initiative, the purpose of which is to provide information and assistance to patients whose cancer treatments have the potential to cause infertility (Fertile Hope, 2011). Fertile Hope’s Sharing Hope Program for Women provides certain fertility medications free of charge and offers one cycle of embryo or oocyte cryopreservation at a discounted rate for eligible patients through partnered reproductive endocrinologists. To be eligible for assistance, annual income must be below $100,000 for a cancer patient who is single and $135,000 for a patient who is married (Fertile Hope, personal communication, February 17, 2012). Even with this assistance, the patient is still responsible for the discounted price of the procedure in addition to other services such as laboratory work and storage fees for frozen eggs or embryos. Financing fertility treatments can be difficult for breast cancer patients who are uninsured, underinsured, or have low or even moderate incomes.

## Fertility Preservation and the AP Role

The ASCO guidelines recommend that patients interested in fertility preservation be referred to a fertility specialist as soon as possible in order to obtain the best outcome. ASCO recognizes embryo cryopreservation as the fertility preservation option most likely to succeed. Data on other methods of fertility preservation are based on case reports, case series, cohort studies, and a few controlled, randomized studies. No increased risk of cancer recurrence has been linked to most fertility preservation options, even with cancers whose growth is known to be stimulated by hormones (Lee et al., 2006).

While the primary concern for most newly diagnosed cancer patients is survival, fertility issues are also important to premenopausal women (Gorman et al., 2011; Redig, Brannigan, Stryker, Woodruff, & Jeruss, 2011). One study demonstrated that for 29% of young women with breast cancer, concern for fertility would influence their treatment decisions (Lee et al., 2011). Most women have little knowledge about the effect of treatment on their fertility. This lack of knowledge increases their decisional conflict (Peate et al., 2011). The research of Lee and colleagues has shown that health-care professionals’ views on fertility options are varied and that conflicting information leads to increased confusion for women who need to make informed choices (2011). Women who are not given sufficient information feel grief at their loss of choice in the matter of fertility preservation. This sense of loss ensues from both a lack of information and information given too late. Patients need to receive information on fertility options that is based on research and not on opinion (Lee et al., 2011).

Less than half of physicians routinely refer premenopausal patients to fertility specialists in accordance with ASCO guidelines (Quinn et al., 2009b). Oncologists’ first priority is the diagnosis and treatment of their cancer patients. As patient volumes increase, less time is available for issues considered less important. Other reasons cited by physicians for not discussing fertility preservation with patients are (1) inadequate knowledge and education about the topic, (2) cultural and language barriers, (3) belief that addressing fertility issues would increase stress, (4) concern about effectiveness of fertility options and the high cost, and (5) hesitation to discuss fertility with patients who have extensive disease (Quinn et al., 2009a).

Although many women have concerns about their fertility, they are often unwilling to broach the topic themselves; therefore, it is imperative that health-care professionals initiate this discussion with their patients (Wilkes, Coulson, Crosland, Rubin, & Stewart, 2010). ASCO now recommends that discussions about fertility preservation occur soon after diagnosis (Lee, 2006). This is particularly important as some approaches to preserving fertility have the potential to delay treatment if not started in a timely manner, and most options cannot be implemented after chemotherapy has been initiated. For women interested in embryo or oocyte cryopreservation, the earlier they are referred to a fertility specialist the more likely it is that they will be able to undergo ovarian stimulation without delay in treatment.

Women with breast cancer may feel that raising fertility issues at the time of initial diagnosis is inappropriate (Jeruss, 2010; Lee at al., 2011). Research shows that discussing fertility the week after diagnosis gives these women time to absorb their condition and consider the implications of treatment (Lee et al., 2011). Oncology advanced practitioners (APs) who work with breast surgeons or medical oncologists are in an ideal position to counsel these women about fertility preservation options. Advanced practitioners should be knowledgeable about interventions for fertility preservation in order to be effective advocates for their patients.

Following a diagnosis of breast cancer it is appropriate for the AP to discuss the impact that cancer treatment will have on fertility. If the patient is interested in pursuing fertility preservation, the AP should explain the various options that are available. Patients can also be referred to support websites such as www.fertilehope.org, www.youngsurvival.org, and www.myoncofertility.org for further information (Hulvat & Jeruss, 2011). Once the patient with breast cancer has made the decision to pursue fertility preservation, the AP should make a timely referral to a fertility specialist.

## Conclusion

In summary, fertility is a major concern for young women diagnosed with breast cancer, second only to survival concerns (Redig et al., 2011). Many of these women receive treatment for their cancer that will have a significant impact on fertility. For women wishing to preserve their fertility, it is important that they are given the appropriate information in a timely manner. It is imperative that APs have an understanding of the fertility preservation options that are available so they can provide accurate information to patients prior to the onset of therapy. Women seeking to pursue one of these treatments need information based on evidence in order to make an informed decision. Advanced practitioners must feel comfortable addressing issues that could be ethically difficult when talking with patients about their options for fertility preservation.

The ASRM recommends fertility assistance for all interested cancer patients and states that the implications of hope, even for patients with poor prognoses, can be far reaching (Ethics Committee of the ASRM, 2005). Advanced practitioners must be advocates for their breast cancer patients. Because loss of fertility can be a devastating result of cancer treatment, APs should initiate a timely referral to a fertility specialist to maximize the chance that treatment will succeed without delaying the initiation of cancer therapy.
